# Letter on “Physiological effects of high-flow nasal cannula oxygen therapy after extubation: a randomized crossover study”

**DOI:** 10.1186/s13613-023-01234-6

**Published:** 2024-01-11

**Authors:** Radhouane Toumi, Emna Ennouri, Mohamed Boussarsar

**Affiliations:** 1 Medical Intensive Care Unit. Research Laboratory N° LR12SP09, Heart Failure, Farhat Hached University Hospital, Sousse, Tunisia; 2 Faculty of Medicine of Sousse, University of Sousse, Sousse, Tunisia

**Dear editor,**

We read with great interest the article by Basoalto et al. entitled “Physiological effects of high-flow nasal cannula oxygen therapy after extubation: a randomized crossover study” published in *Annals of Intensive Care* [1].

The authors studied the physiological effects after extubation within 1 h of high-flow nasal cannula (HFNC) in a crossover physiological study seeking to comprehensively determine HFNC impact in the post-extubation period. We would like to congratulate the authors for the considerable efforts they made to dissect the HFNC mechanisms of action in the post-extubation setting. However, a few reservations are worth considering.

First, we felt that this study lacks a more comprehensive approach to exhaustively study physiological effects in extubated patients. This is particularly noticeable in the choice of patients that, when looking at provided baseline and post-extubation data, appear to be at low risk of extubation failure. In the current era of precision and personalized medicine, it would be more interesting to determine how HFNC potentially benefits certain subgroups or phenotypes of extubated patients with poor respiratory mechanics and at high risk of failure. This is especially true, knowing that it is still not well-determined that HFNC effectively decreases the risk of extubation failure with previous large-scale studies failing to prove so [2, 3]. Also addressing the comprehensive approach, we fail to understand the use of a crossover design in such acute dynamic settings when numerous patient-related factors, such as anxiety and fear with their implications on the neural drive, and bronchiolar and alveolar collapsibility, are constantly changing from one minute to another, let alone over the course of two hours. One of the main mandatory criteria when using crossover design is that the conditions of the subjects must remain stable throughout the study [4]. These patients consequently fail to serve as their own control.

Second, seen the physiological nature of this study, we found ourselves expecting authors to provide us with more than one single measurement at the end of each hour, for instance, closer measures carried-out each 10 min, namely, with electrical impedance tomography. By performing a single measurement after the course of a whole hour, one would fail to detect rapidly occurring dynamic changes, such as with regional lung ventilation potentially resulting in lung recruitment or overdistension, as well as variations with delayed onset, such as muscle impairment or amino-terminal pro-B-type natriuretic peptide variation. Moreover, we were eager to see patient characteristics before extubation, including all performed measurements along with data reflecting respiratory mechanics and viscoelastic properties, such as compliance, peak, plateau, and driving pressures on VC mode. Even more so, we would ideally see data of a second measurement after a stabilization period on pressure support ventilation with pressure support set at 8 cmH2O and positive end-expiratory pressure at zero cmH2O, in addition to last set of physiological measurements, for instance 3 min after extubation on spontaneous breathing, which would also serve to provide true baseline characteristics.

Finally, as stated by the authors, the current findings only allow them to state that muscle unloading is a possible mechanism to avoid extubation failure. Nonetheless, causes for extubation failure vary ranging from high neural drive to increased airway resistance, from laryngospasm or bronchial hyper-reactivity to airway and alveolar collapsibility leading to increased resistance and derecruitment, and to decreased pulmonary compliance, such as in morbidly obese patients or weaning-induced pulmonary edema. The results presented in this study poorly address these issues, mostly because the study population does not appear to have altered neural drive, airway resistance, pulmonary elastance or cardiac function, thus struggling to reach the minimal clinically important difference (MCID). These results and stated conclusions might not be generalizable, perhaps explaining certain discrepancies with previous studies showing HFNC to increase lung recruitment and expiratory end-lung volume [5].

Taking into account the stated considerations, we believe that the determination of certain phenotypes of patients and identifying post-extubation treatable traits within these patients potentially benefiting from HFNC rather than conventional oxygen therapy would be possible and of great value.

## Data Availability

My manuscript has no associated data.

## Abbreviations

HFNC: High flow nasal cannula
VC: Volume control
MCID: Minimal clinically important difference
